# Deep Learning with Neuroimaging and Genomics in Alzheimer’s Disease

**DOI:** 10.3390/ijms22157911

**Published:** 2021-07-24

**Authors:** Eugene Lin, Chieh-Hsin Lin, Hsien-Yuan Lane

**Affiliations:** 1 Department of Biostatistics, University of Washington, Seattle, WA 98195, USA; lines@uw.edu; 2 Department of Electrical & Computer Engineering, University of Washington, Seattle, WA 98195, USA; 3 Graduate Institute of Biomedical Sciences, China Medical University, Taichung 40402, Taiwan; 4 Department of Psychiatry, Kaohsiung Chang Gung Memorial Hospital, Chang Gung University College of Medicine, Kaohsiung 83301, Taiwan; 5 School of Medicine, Chang Gung University, Taoyuan 33302, Taiwan; 6 Department of Psychiatry, China Medical University Hospital, Taichung 40447, Taiwan; 7 Brain Disease Research Center, China Medical University Hospital, Taichung 40447, Taiwan; 8 Department of Psychology, College of Medical and Health Sciences, Asia University, Taichung 41354, Taiwan

**Keywords:** Alzheimer’s disease, artificial intelligence, deep learning, genomics, machine learning, multi-omics, neuroimaging, single nucleotide polymorphisms

## Abstract

A growing body of evidence currently proposes that deep learning approaches can serve as an essential cornerstone for the diagnosis and prediction of Alzheimer’s disease (AD). In light of the latest advancements in neuroimaging and genomics, numerous deep learning models are being exploited to distinguish AD from normal controls and/or to distinguish AD from mild cognitive impairment in recent research studies. In this review, we focus on the latest developments for AD prediction using deep learning techniques in cooperation with the principles of neuroimaging and genomics. First, we narrate various investigations that make use of deep learning algorithms to establish AD prediction using genomics or neuroimaging data. Particularly, we delineate relevant integrative neuroimaging genomics investigations that leverage deep learning methods to forecast AD on the basis of incorporating both neuroimaging and genomics data. Moreover, we outline the limitations as regards to the recent AD investigations of deep learning with neuroimaging and genomics. Finally, we depict a discussion of challenges and directions for future research. The main novelty of this work is that we summarize the major points of these investigations and scrutinize the similarities and differences among these investigations.

## 1. Introduction

Alzheimer’s disease (AD), a progressive brain disorder, is a critical health issue in terms of global health, public health, and population health in the world [1]. Understanding disease progression of AD and facilitating an early diagnosis of AD have been a focus of attention in the fields of neuroimaging and genomics in the past decade [2]. Since 2007, numerous genome-wide association studies (GWASs) have also been performed to identify genetic variants such as single nucleotide polymorphisms (SNPs) associated with AD [3]. Due to the improvements of artificial intelligence (AI) technologies, researchers have continued making notable contributions in the multi-disciplinary fields of machine learning, deep learning, neuroimaging, genomics, and the diagnosis and prediction of AD [4,5]. Up-to-date advances in AI technologies, in particular deep learning techniques, have exhibited their advantageous impacts in connection with health-related and genomic medicine applications [6,7,8,9,10]. For the tasks of the diagnosis and prediction of AD, the goal of computer-aided AI methods such as deep learning models is to facilitate data-driven algorithms that can on the whole help improve the diagnosis accuracy of AD using neuroimaging and/or genomics data [4,5]. For example, deep learning models such as convolutional neural networks (CNNs) have been utilized to detect AD using neuroimaging data such as magnetic resonance imaging (MRI) images [4,5]. Therefore, it has been suggested that deep learning models play a key role in the diagnosis and prediction of AD in the future studies as these promising approaches can be easily applied to numerous data formats with neuroimaging and genomics [4,5].

Deep learning models typically exploit multiple layers of abstraction to determine hierarchical portrayals in the raw data, in which the whole process intends to detect and extract high-level features from the raw data [11,12,13]. In other words, in order to build up the hierarchical representation, deep learning models perform data-driven strategies using deep artificial neural networks with multiple layers, instead of using artificial neural networks with only a single layer [13,14]. Deep learning models have accomplished a wide range of experiments for the tasks of the diagnosis and prediction of AD using the state-of-the-art computing technologies (i.e., general-purpose computing on graphics processing units (GPUs)) [4,5]. For the purpose of handling the challenging difficulties we face currently for the tasks of the diagnosis and prediction of AD, there is an enormous need for utilizing deep learning models such as the CNN model for multiple data types such as neuroimaging and/or genomics data [4,5]. With recent advances in computer software and hardware techniques, deep learning models possess a tremendous potential to be leveraged to identify AD from normal controls and/or to identify AD from mild cognitive impairment [4,5].

Here, in the context of deep learning models, we depict various research studies with the focus on AD prediction using neuroimaging and genomics data. We primarily focus on AD prediction using a wide variety of deep learning models as, to our knowledge, this is one of the hot topics in the multi-disciplinary fields of deep learning, AD prediction, neuroimaging, and genomics. Consequently, biological and/or clinical implications from this topic could then serve as a basis for future research in AD prediction with neuroimaging and genomics using deep learning models. Additionally, we describe the limitations in these research studies and summarize a discussion of future challenges and problems. Although this review does not present the full set of relevant research investigations reported in the literature, it nonetheless indicates a synthesis of those that can markedly contribute to global, public, and population health-oriented studies in AD prediction with neuroimaging and genomics using deep learning models in the near to mid-term future.

## 2. Methods

In this review, we first conducted a comprehensive search of the electronic PubMed and Google Scholar databases (2016-present) using key words such as “deep learning,” “Alzheimer’s disease,” “genomics,” “genetics,” “neuroimaging,” and “imaging”. Then, we manually screened the obtained articles with a specific focus on AD, deep learning models, genomics, neuroimaging, and neuroimaging genomics. Figure S1 presents the preferred reporting items for systematic reviews and meta-analyses (PRISMA) flow diagram based on previous literature [15].

While this review does not intend to cover all related studies in an exhaustive manner, it nevertheless is representative of the general trend for current research on genomics, neuroimaging, and neuroimaging genomics for AD prediction using deep learning models.

## 3. Deep Learning Models

The initial concept of deep learning models has a long history dating back at least two decades with an aim to use the idea of multiple processing layers (i.e., multiple levels of abstraction) [6]. However, it was infeasible for large-scale applications in the beginning due to the intense computational time needed to perform the task. After the advent of GPUs around 2009, multiple processing layers in deep learning models can then be computed at least 10 to 20 times faster [16]. Since then, deep learning models have become a focus of attention in a wide variety of research areas. Remarkably, deep learning models have become state of the art in the fields of computer vision and natural language processing where enormous accomplishments have been achieved [17,18]. Due to the fact that a flood of investigations have been conducted using a wide variety of variants of deep learning models in various fields of science and engineering, it is indeed challenging to catch up with the emerging direction [6].

Deep learning models provide the following merits. Firstly, in reference to the empirical experiments, deep learning models usually offer better performance than the traditional machine learning approaches [6]. Secondly, deep learning models can perform sampling tasks concurrently. As a result, they contribute to a significant speedup for processing big data (i.e., large-scale applications) [6]. Thirdly, deep learning models can process natural data in their original form without transforming them into internal representations [6]. Moreover, deep learning models can normally provide the functionality of feature extraction in the deep learning model itself [4].

### 3.1. Applications of Deep Learning Models

Deep learning models can be applied to a wide range of research areas, including computer vision, image processing, natural language processing, speech processing, and video processing, to name a few [6,17,18,19,20]. Additionally, deep learning models have been implemented in biomedical applications such as genomics, multi-omics, and medical imaging [21,22,23,24].

### 3.2. Variants of Deep Learning Models

**Figure 1:**
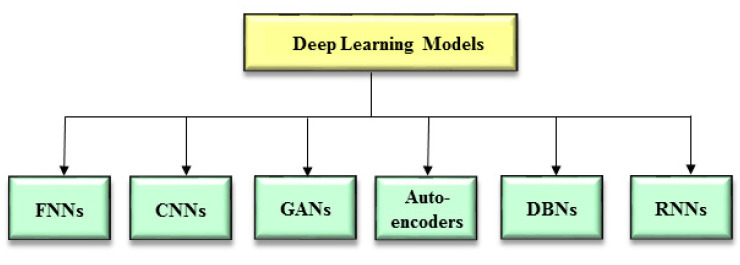
Variants of deep learning models. CNNs = Convolutional Neural Networks; DBNs = Deep belief networks; FNN = Fully Connected Neural Networks; GANs = Generative Adversarial Networks; RNNs = Recurrent Neural Networks.

There are a wide variety of deep learning-based models (namely variants of deep learning models) (Figure 1). In this subsection, we introduce the following six variants: fully connected neural networks (FNNs), convolutional neural networks (CNNs), generative adversarial networks (GANs), auto-encoders, deep belief networks (DBNs), and recurrent neural networks (RNNs). These variants were employed by the investigations which are depicted in this review. In addition, the reader can refer to recent reviews by Shrestha et al. [19] and Zhou, S.K. et al. [21] for other variants of deep learning models.

#### 3.2.1. Fully Connected Neural Networks (FNNs)

The FNN model is the foundation of deep learning models that mainly comprise of fully connected (or dense) layers, where every unit/neuron in one layer is connected to every unit/neuron in the next layer [25].

#### 3.2.2. Convolutional Neural Networks (CNNs)

The CNN model is a branch of deep learning models which mainly comprise of an input layer, hidden layers, and an output layer [26,27]. In the CNN model, there are three main types of layers: convolutional layers, pooling layers, and fully connected layers [26,27]. The convolutional layers are able to extract various features from an image. The pooling layers are suitable for reducing the size of the feature representation. The fully connected layers are capable of providing the non-linear combinations of the feature representations. Initially, the CNN model was applied to the tasks of object recognition and classification for image, speech, or audio signal input in the fields of computer vision and natural language processing [26,27].

The residual CNN model, a variant of CNN, adopts a residual function to obtain residual representations/vectors and thereby to gain improved performance [28]. In computer vision applications, it is more effective to process residual representations/vectors than the original representations/vectors [28].

#### 3.2.3. Generative Adversarial Networks (GANs)

Goodfellow et al. [29] created the GAN model to perform an adversarial function using a form of generative models. Subsequently, the GAN model has become one of most prominent research areas in deep learning and is mainly applied in the field of computer vision and image processing (such as image generation) [30,31].

In brief, the GAN model consists of two key components, namely a generative network and a discriminative network [29]. While the generative network is trained to produce fake data in compliance with a latent variable, the discriminative network acquires both real and fake data to decide whether the data is real or not. Both the generative and discriminative networks accomplish an adversarial game against each other at the same time.

In order to cope with the instability of GAN training, Arjovsky et al. [32] proposed the Wasserstein GAN model which utilizes a new distance metric function called the Earth-Mover distance (i.e., Wasserstein distance). On the other hand, the original GAN model employs the Jensen–Shannon divergence, which is theoretically impractical to evaluate the distance between two distributions if the distributions do not overlap [32].

Additionally, a variant of the Wasserstein GAN model called the conditional Wasserstein GAN model is essentially portrayed as the Wasserstein GAN model by adding a gradient penalty term to obtain enhanced performance [32,33].

#### 3.2.4. Auto-Encoders

In essence, the auto-encoder model consists of an encoder unit and a decoder unit, where these two units are generally implemented using fully connected layers.

The variational auto-encoder model [34] exemplifies a variant of the auto-encoder model. Fundamentally, the variational auto-encoder model only comprises an encoder unit and a decoder unit, without an adversarial network. The training goal of the encoder unit is to produce the mean and covariance of the Gaussian distribution to portray the variational distribution in the variational auto-encoder model [34]. Note that the variational auto-encoder model is not a variant of the GAN model.

The adversarial auto-encoder model [35] also exemplifies a variant of the autoencoder model and represents a probabilistic/generative model. Essentially, the adversarial auto-encoder model consists of a conventional auto-encoder and an adversarial network. In general, the training goal in the adversarial auto-encoder model is to match the latent data produced by the generative network module with a specific prior latent distribution. Note that the adversarial auto-encoder model is a variant of the GAN model. The adversarial auto-encoder model can be combined with the variational auto-encoder model to form the adversarial variational auto-encoder model [36].

These three auto-encoder-based models (i.e., the variational auto-encoder, adversarial auto-encoder, and adversarial variational auto-encoder models) can be used as benchmarking algorithms when applying the auto-encoder model. It should be note that these three auto-encoder-based models are not the focus of this review.

#### 3.2.5. Deep Belief Networks (DBNs)

In the field of deep learning, the DBN model is a branch of deep learning models that comprise of multiple layers of hidden/latent variables, and each layer gathers the correlation information among the activities of hidden/latent variables in the previous layer [37,38]. The building block for each layer can be a restricted Boltzmann machine, which is a two-way undirected graphical model [37,38].

A variant of DBN, referred to as a sparse-response deep belief network, was proposed by adopting the rate distortion theory, which encodes the original data into a sparse dataset with few bits to obtain better performance [39].

#### 3.2.6. Recurrent Neural Networks (RNNs)

In the field of deep learning, the RNN model is a branch of deep learning models, which is capable of processing time series measurements or sequential data such as DNA sequences [22]. RNNs may need more computing time than CNNs as it is difficult for them to perform in parallel [22].

## 4. Research Studies in Genomics on the Prediction of AD Using Deep Learning

In this section, we focus especially on the prediction of AD with genomics data using deep learning models. The usage of the deep learning approach is still in its infancy in the context of AD prediction with genomics data. Lately, deep learning-based applications in AD prediction with genomics data have experienced some revival in the field of artificial intelligence and machine learning. Here, we focus on AD prediction with genomics data using various deep learning models in this section (Table 1 and Table 2).

The genomics/multi-omics data in these studies included SNPs datasets, DNA methylation datasets, and gene expression datasets. While this review does not intend to depict all existing investigations in an exhaustive way, it still is representative of the present trend for research in AD prediction with genomics data using deep learning models. In addition, the reader can refer to a review by Eraslan et al. [22] and Zou et al. [23] for other applications in genomics using deep learning models.

As described in the following subsections, there are some studies for AD research with genomics data using various deep learning models (Table 1), including the prediction of AD risk, the prediction of AD-specific nucleotide alteration sites (i.e., splicing sites), and the prediction of the virtual disease/molecular progress of AD.

### 4.1. Integrated Deep Learning and Machine Learning Approach with Gene Expression

Maj et al. [40] proposed an integrated deep learning and machine learning approach to analyze gene expression data in connection with AD cognitive decline. Their integrated approach leverages the concept of variational auto-encoders (see Section 3.2.4.), FNNs (see Section 3.2.1.), CNNs (see Section 3.2.2.), and RNNs (see Section 3.2.6.): the first model was used for the purpose of features selection; the latter three models were implemented to analyze temporal gene expression data [40]. The integrated approach also employs six sample tissues, such as adipose subcutaneous, artery aorta, colon transverse, brain spina, thyroid, and whole blood tissues [40]. In addition, the support vector machine classifier was used to estimate if the input dataset to the variational auto-encoder aligned with a particular tissue [40].

Their analysis revealed that the RNN model outperformed FNNs and CNNs on the adipose subcutaneous (the area under the curve (AUC) = 0.953, 0.513, and 0.948, respectively), artery aorta (AUC = 0.951, 0.503, and 0.862, respectively), and colon transverse (AUC = 0.946, 0.862, and 0.770, respectively) tissues [40]. On the other hand, the CNN model exceeded FNNs and RNNs on the brain spina (AUC = 0.943, 0.892, and 0.942, respectively), thyroid (AUC = 0.95, 0.503, and 0.946, respectively), and whole blood (AUC = 0.947, 0.516, and 0.939, respectively) tissues [40].

The main drawback of the study by Maj et al. [40] is that only one genomic component, namely gene expression, was considered and thereby other potential genetic effects may be overlooked. The second drawback is that one cannot draw conclusive results due to the small sample size of the gene expression profiles [40]. The third drawback is that sex-specific organs/tissues should be investigated because of the different prevalence rate of AD in female and male populations [40].

On the other hand, the main merit of their study is that their integrated approach utilized state-of-the-art deep learning models, such as FNNs, CNNs, and RNNs, as benchmarking models to demonstrate its performance.

### 4.2. Fully Connected Neural Networks with Gene Expression

Lee and Lee [41] utilized a deep learning-based model to predict AD risk using gene expression data. Their deep learning-based model was based on the concept of FNNs (see Section 3.2.1.). In line with Maj et al. [40], variational auto-encoders were used for feature selection to extract a representation from gene expression data in three blood gene expression datasets. In addition, a differentially expressed gene (DEG)-based method was used for feature selection for comparison. In their study, various machine learning methods, such as logistic regression, L1-logistic regression, support vector machines, and random forests, were utilized for benchmarking.

Their data showed that the models with the DEG-based feature selection method surpassed the ones with variational auto-encoders as variational auto-encoders may not acquire the key information [41]. Moreover, the deep learning-based model did not always excel other benchmarking models in all cases of three datasets as deep learning is infeasible for the small sample size of instances, which consist of high dimensional information (i.e., a large number of genes) [41]. Note that for the previous statements, no specific values for accuracy/AUC (i.e., only figures) were reported in their study.

The main disadvantage of the study by Lee and Lee [41] is that only one genomic component, namely gene expression, was examined and thereby other likely genetic impacts may be disregarded. The second disadvantage is that one cannot draw sound conclusions due to the small sample size of the gene expression profiles.

On the other hand, the main advantage of their study is that their approach was compared to various state-of-the-art machine learning models, such as logistic regression, L1-logistic regression, support vector machines, and random forests, to demonstrate its performance.

### 4.3. GANs with Gene Expression

Park, J. et al. [42] used a deep learning-based model to predict the virtual disease/molecular progress of AD using gene expression data from the AD model of mouse data. Their deep learning-based model was characterized by the concept of GANs, namely the Wasserstein GAN model with a gradient penalty term [32,33] (see Section 3.2.3.). The latent space interpolation (i.e., vector arithmetic) of GANs was leveraged to describe pathological pathway cascades in the AD disease progression. In addition, only 1208 DEGs were selected as training data for the purpose of the pathological pathway analysis due to the small number of samples with the burden of high dimensional information (i.e., a large number of genes).

Their results suggested that cholesterol biosynthesis is initiated during an early stage of AD and is triggered by amyloid-beta production [42]. It has been reported that interactions between cholesterol biosynthesis and amyloid-beta production may contribute to synapse plasticity [51].

The main limitation of the study by Park, J. et al. [42] is that one cannot draw sound conclusions due to too few genes and the small sample size of the gene expression profiles. More genes and more high-quality augmentation data are warranted in the future studies. Moreover, another limitation is that Park, J. et al. [42] did not compare their proposed model with other existing models to demonstrate its performance.

On the other hand, the main benefit of their study is that their approach was the first to leverage the concept of GANs to provide the virtual disease/molecular progress of AD using gene expression data.

### 4.4. Residual CNN with Gene Expression

Kim et al. [43] employed a deep learning-based framework called SpliceAI [52] to predict AD-specific nucleotide alteration sites (i.e., splicing sites) using pre-messenger RNA (mRNA) nucleotide sequences. The SpliceAI framework was built on a variant of CNNs called the residual CNN model [28] (see Section 3.2.2.), which is frequently used for computer vision applications.

Their SpliceAI analysis predicted 14 splicing sites in the *PLCG1* gene and the AD-associated single-nucleotide variants (SNVs) occurred at the same position in *PLCG1* in humans and in the AD mouse model cortex [43]. While it has been suggested that the *PLCG1* gene is associated with AD risk [53], it is interesting that Kim et al. [43] identified SNVs in the *PLCG1* gene associated with AD using a deep learning technique.

The main weakness of the study by Kim et al. [43] is that one cannot draw sound conclusions as only one gene (i.e., *PLCG1*) was investigated as a pilot study using a deep learning approach. More genes and more high-quality gene expression data are warranted in the future studies. Moreover, another weakness is that Kim et al. [43] did not compare their proposed model with other existing models to demonstrate its performance.

On the other hand, the main strength of their study is that their approach was the first to adopt the concept of the residual CNN model to provide AD-specific SNVs using gene expression data.

### 4.5. Fully Connected Neural Networks with Gene Expression and DNA Methylation

Park, C. et al. [44] implemented a deep learning-based model to predict AD risk using an integrated data of gene expression and DNA methylation. Their deep learning-based model was adapted from the concept of FNNs (see Section 3.2.1.), where Bayesian optimization was performed for hyper-parameter search. In their study, various machine learning methods, such as naïve Bayesian, support vector machines, and random forests, were utilized for benchmarking. Park, C. et al. [44] also proposed a differentially expressed gene-based and differentially methylated position-based approach for feature selection to reduce the dimensions/features. In addition, conventional dimension reduction methods such as principal component analysis and t-distributed stochastic neighbor embedding were used for comparison.

Their findings demonstrated that the deep learning-based model (AUC = 0.797) outperformed various benchmarking models such as naive Bayesian (AUC = 0.756), support vector machines (AUC = 0.773), and random forests (AUC = 0.775) [44]. Additionally, the differentially expressed gene-based and differentially methylated position-based approach (AUC = 0.797) also exceeded principle component analysis (PCA) (AUC = 0.612) and t-stochastic nearest neighbor (t-SNE) (AUC = 0.526) [44]. Moreover, the performance for the integrated data of gene expression and DNA methylation was better than the one for gene expression alone or DNA methylation alone [44]. Note that for the previous statement, no specific values for accuracy/AUC (i.e., only figures) were reported in their study.

The main drawback of the study by Park, C. et al. [44] is that one cannot draw conclusive results due to the small sample size of the integrated gene expression and DNA methylation profiles. Second, the proposed model may be over-fitted during the data oversampling procedure. Moreover, the fundamental classifier, namely logistic regression, was not used for benchmarking. In addition, other deep learning algorithms such as CNNs and RNNs were not utilized for comparison.

On the other hand, the main merit of their study is that two types of multi-omics data (i.e., gene expression and DNA methylation) were integrated to establish prediction models, which showed improved performance. Second, their deep learning-based model was compared with various state-of-the-art machine learning models, such as naïve Bayesian, support vector machines, and random forests, to demonstrate its superior performance.

## 5. Research Studies in Neuroimaging on the Prediction of AD Using Deep Learning

In this section, we focus especially on the problem of AD prediction with neuroimaging data using deep learning models. The usage of the deep learning approach in terms of predicting AD has been a focus of attention in neuroimaging research. Lately, deep learning-based applications in AD prediction with neuroimaging data have experienced some revival in the field of artificial intelligence and machine learning. Here, we focus on AD prediction with neuroimaging data using various deep learning models in this section (Table 1 and Table 2).

Several notable examples of open source databases for neuroimaging in AD include the Alzheimer’s Disease Neuroimaging Initiative (ADNI) (adni.loni.usc.edu, accessed on 20 June 2021) [2], the Australian Imaging, Biomarker & Lifestyle Flagship Study of Ageing (AIBL) (aibl.csiro.au, accessed on 20 June 2021) [54], the Open Access Series of Imaging Studies (OASIS) (www.oasis-brains.org, accessed on 20 June 2021) [55], the Japanese Alzheimer’s Disease Neuroimaging Initiative (J-ADNI) (jadni.com, accessed on 20 June 2021) [56], and the Worldwide Alzheimer’s Disease Neuroimaging Initiative (WW-ADNI) [57], to name a few.

Predicting AD with neuroimaging data has been a focus of attention for at least two decades, from employing the conventional machine learning methods (such as support vector machines and ensemble methods) to using deep learning approaches (such as CNNs) [4]. While this review does not intend to depict all existing investigations in an exhaustive way, it still is representative of the present trend for research in AD prediction with neuroimaging data using deep learning models. In addition, the reader can refer to a recent review by Tanveer et al. [4], which listed at least 165 research reports from 2005 to 2019 regarding machine learning and deep learning approaches in AD prediction with neuroimaging data.

There are a wide variety of neuroimaging modalities for determining the predictive features of AD, including structural MRI, diffusion tensor imaging, electroencephalography, functional MRI, positron emission tomography (PET), single-photon emission computed tomography, near-infrared spectroscopy, and proton magnetic resonance spectroscopy [58,59].

### 5.1. Auto-Encoders with MRI Images

For instance, Ju et al. [45] used a deep learning-based approach to predict early diagnosis of AD using MRI data. Their deep learning approach was established on a softmax regression layer and auto-encoders (see Section 3.2.4). Basically, an auto-encoder is an artificial neural network for the encoding purpose, where the input layer serves the original MRI data, multiple hidden layers provide nonlinear transformations from the previous layers, and the output layer reconstructs MRI samples. In their study, various widely used machine learning methods, such as linear discriminant analysis, logistic regression, and support vector machines, were utilized for benchmarking.

Ju et al. [45] reported that the proposed auto-encoder method (AUC = 0.916) surpassed various benchmarking models such as linear discriminant analysis (AUC = 0.710), logistic regression (AUC = 0.765), and support vector machines (AUC = 0.789) to predict an early stage of AD using MRI images.

The main disadvantage of the study by Ju et al. [45] is that only single-modal brain imaging data, namely MRI images, was examined and thereby other likely morphological changes from multimodal brain image data may be disregarded. Moreover, other deep learning algorithms such as CNNs and other machine learning algorithms such as random forests were not utilized for comparison.

On the other hand, the main advantage of their study is that their approach was the first to exploit the concept of auto-encoders to predict early diagnosis of AD using MRI data.

### 5.2. Deep Belief Networks with PET Images

Shen et al. [46] employed a deep learning-based approach to distinguish AD from mild cognitive impairment using PET data. Their deep learning approach was based on DBNs (see Section 3.2.5.), which serves as feature selection to identify key features from the regions of interest (ROIs). The support vector machine model, which is a popular machine learning method for detecting AD with structural MRI data, was utilized to distinguish AD from mild cognitive impairment.

Shen et al. [46] discovered that the proposed DBN-based method obtained good performances for differentiating subjects between AD and mild cognitive impairment (AUC = 0.908). In addition, the DBN model (accuracy = 0.866) excelled PCA (accuracy = 0.795) and anatomical automatic labeling (accuracy = 0.631) [46].

The main limitation of the study by Shen et al. [46] is that only single-modal brain imaging data, namely PET images, was examined and thereby other likely morphological changes from multimodal brain image data may be disregarded. Moreover, other deep learning algorithms such as CNNs and other machine learning algorithms such as random forests were not utilized for comparison. Their study also did not always use AUC, a standard evaluation metric, for comparison.

On the other hand, the main benefit of their study is that their approach was the first to apply the concept of DBNs to distinguish AD from mild cognitive impairment using PET data.

### 5.3. Sparse-Response Deep Belief Networks with PET and MRI Images

Zhou, P. et al. [47] utilized a deep learning-based approach to predict AD using PET and MRI images. Their deep learning approach was characterized by sparse-response DBNs [39] (see Section 3.2.5), which was used for extracting features from the images. Then, the extreme learning machine model was utilized to distinguish AD, mild cognitive impairment, and normal controls. In their study, support vector machines and CNNs were utilized for benchmarking.

Zhou, P. et al. [47] indicated that the proposed approach (AUC = 0.87) outperformed the benchmarking models, such as CNNs (AUC = 0.77) and DBNs (AUC = 0.83) for distinguishing AD and normal controls. In addition, the proposed approach (AUC = 0.79) exceeded CNNs (AUC = 0.60) and DBNs (AUC = 0.73) for distinguishing mild cognitive impairment and normal controls [47]. Moreover, the proposed approach (AUC = 0.71) surpassed CNNs (AUC = 0.60) and DBNs (AUC = 0.68) for distinguishing AD and mild cognitive impairment [47].

The main weakness of the study by Zhou, P. et al. [47] is that other deep learning algorithms such as GANs and other machine learning algorithms such as random forests were not utilized for comparison.

On the other hand, the main strength of their study is that their approach was the first to leverage the concept of sparse-response DBNs to predict AD using multimodal brain image data such as PET and MRI images.

## 6. Research Studies in Neuroimaging Genomics on the Prediction of AD Using Deep Learning

**Figure 2:**
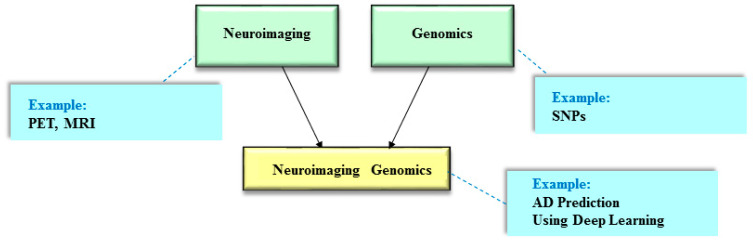
The integrated approach of neuroimaging and genomics. The idea of integrating neuroimaging and genomics is designated as neuroimaging genomics. Deep learning models with neuroimaging genomics can be used to predict Alzheimer’s disease (AD) risk. MRI = magnetic resonance imaging; PET = positron emission tomography; SNPs = Single nucleotide polymorphisms.

The initial concept of incorporating neuroimaging and genomics has a long history dating back to at least two decades ago with an aim to use the idea of intermediate endophenotypes by integrating the strengths of both neuroimaging and genomics studies [60,61] (Figure 2). In the past, this ensemble approach is known as imaging genomics or the interchangeable term, namely imaging genetics. Nowadays, it is also known as neuroimaging genomics, which exemplifies the intersection of the two fields, neuroimaging and genomics [62]. A notable example of neuroimaging genomics is the Enhancing NeuroImaging Genetics through Meta-Analysis Consortium (ENIGMA) [63], which aims to understand how psychiatric disorders influence the brain by combining neuroimaging (e.g., MRI, diffusion tensor imaging, and functional MRI) and genomics (e.g., GWAS) data.

In neuroimaging, the notion of intermediate endophenotypes was considered to offer quantitative measures for behavioral phenotypes; thereby possibly generating a connection between genes and behavioral phenotypes [64,65]. In short, the intermediate endophenotypes are the quantitative biomarkers of brain activities captured by neuroimaging modalities and can be leveraged to evaluate neurobiological alterations of brain functions affected by brain disorders such as AD [66]. It is suggested that AD-related genomic variants might manifest in the intermediate endophenotypes that can be assessed using neuroimaging modalities and may be directly involved with AD [67,68]. In addition, it is indicated that the intermediate endophenotypes of neuroimaging can be used to tackle the issue of small effect sizes in genomics studies [67,68]. On the contrary, it is implicated that there are only a handful of replicated studies in the existing findings of neuroimaging genomics, which might be owing to the limited sample sizes, unreliable study designs, and a lack of corrections for multiple testing [69,70].

In this section, we focus especially on the issue of AD prediction with neuroimaging genomics using deep learning models. In the current literature, such studies in the joint investigation of neuroimaging and genomics (i.e., neuroimaging genomics; Figure 2) are still rare, especially for AD prediction. Here, we focus on AD prediction with neuroimaging genomics using various deep learning models in this section (Table 1 and Table 2).

### 6.1. Fully Connected Neural Networks with Genetic Variants and MRI-Derived Brain Measures

Ning et al. [48] proposed a deep learning-based model to predict AD risk using genetic variants (i.e., SNPs) and neuroimaging data (i.e., MRI-derived brain morphometric measures). Their deep learning-based model was built on the concept of FNNs (see Section 3.2.1). Here, brain morphometric measures are defined as the volume measurements of candidate brain regions in imaging modalities (e.g., MRI) that might be associated with AD. In addition, the logistic regression model was used as a basis for comparison.

Their analysis revealed that the performance for the integrated data of SNPs and MRI-derived brain morphometric measures (AUC = 0.948) was superior to the one for SNP alone (AUC = 0.689) or MRI-derived brain morphometric measures alone (AUC = 0.820) [48]. Moreover, the proposed deep learning-based model (AUC = 0.948) excelled the logistic regression model (AUC = 0.945) for distinguishing AD and normal controls using the integrated data of SNPs and MRI-derived brain morphometric measures [48]. Furthermore, the proposed deep learning-based model (AUC = 0.846) outperformed the logistic regression model (AUC = 0.824) for distinguishing AD and mild cognitive impairment using the integrated data [48].

The main drawback of the study by Ning et al. [48] is that only single-modal brain imaging data, namely MRI, was examined and thereby other likely morphological changes from multimodal brain image data may be disregarded. Likewise, only one genomic component, namely SNPs, was considered and thereby other potential genomic effects may be overlooked. Moreover, other deep learning algorithms such as DBNs and other machine learning algorithms such as random forests were not utilized for comparison.

On the other hand, the main merit of their study is that their approach was the first to adopt the concept of FNNs to predict AD using SNPs and MRI-derived brain morphometric measures.

### 6.2. Three-Stage FNNs with Genetic Variants, PET, and MRI

Zhou, T. et al. [49] used a deep learning-based model to forecast AD risk using genetic variants (i.e., SNPs) and neuroimaging data (i.e., the regions of interest (ROIs) in PET and MRI images). Their deep learning-based model was adapted from the concept of FNNs (see Section 3.2.1), where FNNs were implemented using a three-stage architecture. Here, the ROIs are defined as candidate brain regions/features in imaging modalities (such as MRI) that might be associated with AD. In addition, the support vector machine algorithm was used as a benchmarking machine learning model.

Their data showed that the performance for the integrated data of SNPs and ROIs (in PET + MRI) was in excess of the one for SNP alone, ROIs in PET alone, ROIs in MRI alone, or other combinations (i.e., SNPs + PET, SNPs + MRI, or PET + MRI) [49]. Moreover, the proposed deep learning-based model exceeded the support vector machine algorithm [49]. Note that for the previous statements, no specific values for accuracy/AUC (i.e., only figures) were reported in their study.

The main disadvantage of the study by Zhou, T. et al. [49] is that only one genomic component, namely SNPs, was considered and thereby other potential genomic effects may be overlooked. Moreover, other deep learning algorithms such as DBNs and other machine learning algorithms such as random forests were not utilized for comparison. Their study also did not use AUC, a standard evaluation metric, for comparison.

On the other hand, the main advantage of their study is that their approach was the first to exploit the concept of three-stage FNNs to predict AD using SNPs, PET, and MRI data.

### 6.3. CNNs with Genetic Variants and MRI

Zhou, J. et al. [50] employed a deep learning-based model to perform the prediction of AD risk using genetic variants (i.e., SNPs) and neuroimaging data (i.e., ROIs in MRI). Their deep learning-based model was established on the concept of CNNs (see Section 3.2.2.). In addition, the random forests algorithm was used for feature selection to reduce the dimensions/features. In their study, various machine learning methods, such as multivariate regression, ridge regression, group-sparse multi-task regression and feature selection [71], and multi-task feature learning [72,73], were utilized for benchmarking.

Their results suggested that the performance for the integrated data of SNPs and ROIs (AUC = 0.8) was better than the one for SNP-only (AUC = 0.6) or ROIs-only (AUC = 0.77) [50]. Moreover, the proposed deep learning-based model surpassed various benchmarking machine learning models such as multivariate regression, ridge regression, group-sparse multi-task regression and feature selection, and multi-task feature learning [50]. Note that for the previous statement, no specific values for accuracy/AUC (i.e., only figures) were reported in their study.

The main limitation of the study by Zhou, J. et al. [50] is that only single-modal brain imaging data, namely MRI, was examined and thereby other likely morphological changes from multimodal brain image data may be disregarded. Likewise, only one genomic component, namely SNPs, was considered and thereby other potential genomic effects may be overlooked. Moreover, other deep learning algorithms such as DBNs and other machine learning algorithms such as support vector machines were not utilized for comparison.

On the other hand, the main benefit of their study is that their approach was the first to apply the concept of CNNs to predict AD using SNPs and MRI data.

## 7. Summaries and Limitations

In summary, the investigations as depicted in the previous sections have some similarities and differences. First, these investigations employed various deep learning-based models (e.g., FNNs, CNNs, GANs, auto-encoders, DBNs, and RNNs) to perform various tasks in AD prediction. Second, these investigations utilized various neuroimaging and genomics data, for example, gene expression and/or DNA methylation in Section 4, MRI and/or PET in Section 5, as well as SNPs, MRI, and/or PET in Section 6. Moreover, most of these investigations used the ADNI database, except three studies (which used the Gene Expression Omnibus database). However, those studies using the ADNI database had different sample sizes (varied from 109 to 805; Table 2). Additionally, most of these investigations applied the cross-validation procedures except three studies (Table 2). However, those studies using the cross-validation procedures had different fold numbers (e.g., 5-fold, 10-fold, and 20-fold). Based on the performance results of these investigations, most of the proposed deep learning-based models outperformed the traditional machine learning approaches except one study by Lee and Lee [41] (Table 2). Only two studies by Maj et al. [40] and by Zhou, P. et al. [47] compared the proposed deep learning-based models to other deep learning models. For example, Zhou, P. et al. [47] suggested that the sparse-response DBN-based model exceeded CNNs in differentiating AD and normal controls, differentiating MCI and normal controls, as well as differentiating AD and MCI. Maj et al. [40] also reported that RNNs outperformed CNNs and FNNs on certain tissues (i.e., adipose subcutaneous, artery aorta, and colon transverse tissues), and CNNs outperformed RNNs and FNNs on other tissues (i.e., brain spina, thyroid, and whole blood tissues). It should be noted that it appears to be difficult to explain which deep learning-based model in these studies produces the best results as none of these investigations leveraged universal benchmark databases and standardized benchmark models, which are discussed in the following limitations section.

In the investigations as depicted in the previous sections, only one study by Zhou, P. et al. [47] reported the trade-offs compared to other methods in terms of the computational complexity of the models. That is, Zhou, P. et al. [47] indicated that the proposed sparse-response DBNs-based approach was faster that CNNs and DBNs (i.e., computational time/cost = 36.2, 1386.5, and 491.7 s, respectively). In addition, none of these investigations reported the trade-offs compared to other methods in terms of the memory/space cost. It has also been suggested that RNNs perform much slower than CNNs as RNNs require sequential operations [22]. However, a lack of standard evaluation criteria (e.g., universal benchmark databases and standardized benchmark models) may lead to doubtful trade-offs [74].

The investigations as depicted in the previous sections should be clarified by considering various disadvantages of the aforementioned research studies in the interdisciplinary fields of the diagnosis and prediction of AD, neuroimaging, genomics, and deep learning. One primary limitation of these previous findings is that there were no comprehensible conclusions as universal benchmark databases were unavailable at the time of publication to thoroughly perform sound comparisons between various deep learning and machine learning models in the past investigations [75,76]. Since standardized benchmark databases have led to rapid progresses in the field of computer vision, it is believed that the field of the diagnosis and prediction of AD can also reap the benefit of standardized benchmark databases [8]. While there are several open-source databases available for neuroimaging and genomics in AD such as ADNI, AIBL, OASIS, J-ADNI, WW-ADNI, and ENIGMA, there are currently no universal benchmark databases to perform reproducible evaluation for the previous findings [77]. Moreover, it is difficult to compare different neuroimaging studies even when the same model (e.g., the CNN model) was used as these studies employed distinct approaches in terms of sample sizes, image preprocessing pipelines (i.e., image deformation), feature extraction, cross-validation, and evaluation metrics [77,78,79]. Therefore, universal benchmark databases are crucially needed for the diagnosis and prediction of AD.

In addition, lacking comparison with other existing models is a common limitation for the aforementioned research studies. It is critical for the aforementioned research studies to compare the proposed methods to standardized benchmark models, which allow for easy comparison. It is also critical for the aforementioned research studies to generalize the findings with independent samples using standardized benchmark databases, which again allow for easy comparison [75,76]. As mentioned previously, it is an open challenge to provide large-scale benchmark databases [80,81] for subsequent analyses in deep learning experiments. Therefore, future deep learning research should be reliably reproducible in compliance with commonly well-accepted benchmark models and large-scale benchmark databases, which should be fulfilled by the research community in the interdisciplinary fields of the diagnosis and prediction of AD, neuroimaging, genomics, and deep learning.

Another typical limitation is that the aforementioned studies may not make use of the cross-validation procedures to prevent the risk of overfitting during the training and testing processes [8]. Over-fitting is a common issue for the MRI image datasets due to the small sample size [78]. It is critical for the aforementioned research studies to perform standardized cross-validation procedures, which allow for easy comparison. Commonly, appropriate procedures include the repeated 10-fold cross-validation procedure and leave-one-out cross-validation procedure for scrutinizing the generalization of deep learning models [82,83,84,85]. In short, the repeated 10-fold cross-validation procedure randomly splits up the whole cohort into ten subdivisions, and then the deep learning models can be trained using 9/10 of the subdivisions and tested using the remaining tenth of the subdivisions [86]. Next, the previous step is repeated nine more times using alternative 9/10 of the subdivisions for training and an alternative tenth of the subdivisions for testing. In similar fashion, the leave-one-out cross-validation procedure is an extreme circumstance where the number of folds is equivalent to the number of samples in the entire cohort [87]. The leave-one-out cross-validation procedure is generally leveraged when the number of samples in the whole cohort or in a particular subdivision is limited [87]. In summary, we assume that the cross-validation procedures may presumably be attributed to the overall performances and configurations for the consequent deep learning models [88].

Yet, another typical limitation in the aforementioned studies is that these studies may not employ the conventional linear models (e.g., linear regression or logistic regression) as the basic foundation for comparison. It should be pointed out that we should make use of the conventional linear models as the underlying basis when we utilize deep learning algorithms such as the CNN model [89]. That is, deep learning models such as the CNN model should be a compatible model not only to the conventional linear models but also to non-linear machine learning models such as random forests and support vector machines. The support vector machine model has been one of the most widely used machine learning algorithms for AD prediction as it provides global optima [4]. On the other hand, a drawback of deep learning models (e.g., FNNs) is the issue of local minima [4].

In terms of the GAN model itself, two major limitations include difficulty in training and the mode collapse problem: the former limitation is that the convergence of the GAN model is substantially unstable and laborious during the training process [90]; the latter limitation is that the GAN model is infeasible to handle the multimodal distributions of the real data during the training process [91].

It should be emphasized that the following limitations may be caused by not only deep learning models but also the conventional machine learning algorithms. First, the performance of deep learning models would be fairly inadequate due to the small sample size and high dimensionality of neuroimaging/genomics data [31]. Second, image preprocessing is a critical issue (i.e., computer hardware limitations) as it requires intense computational resources (e.g., GPUs and memory) [5]. Yet, another limitation is that it is particularly challenging to comprehend the underlying meaning of deep learning models. Currently, deep learning models such as the CNN model are viewed as “black boxes”, which are usually problematic to interpret and thereby cause issues in the field of healthcare, especially [92]. It is evident that inherently interpretable deep learning models, instead of black boxes, are warranted [92]; therefore, we could determine explainable characteristics identified by deep learning models.

It should be noted that the following open challenges and emerging problems may be attributed to not only deep learning models but also the conventional machine learning algorithms. In terms of the diagnosis and prediction of AD with neuroimaging and genomics using deep learning models, one main open challenge and emerging problem is that open-source software platforms/packages are critically warranted because of importance in software replicability and reusability [93]. Secondly, universal benchmarking frameworks (e.g., benchmark databases, benchmark models, image preprocessing pipelines, and cross-validation procedures) are lacking and challenging to date in the diagnosis and prediction of AD with neuroimaging and genomics using deep learning models. Thirdly, it could be very challenging and problematic to compare a proposed model with other exiting deep learning and machine learning models, which is often lacking in the aforementioned research studies.

In terms of the GAN model itself, two main open challenges and emerging problems include the instability issue and the mode collapse issue, as mentioned previously. The former one is to resolve the instability issue in the generative and discriminative network modules, where the cost functions constantly fail to converge during the training process [90]. The latter is to tackle the mode collapse issue, in particular when multimodal distributions are extraordinarily sophisticated in the real data [91].

Finally, only a handful of investigations have been conducted to date by using deep learning models in the interdisciplinary fields of the diagnosis and prediction of AD, neuroimaging genomics, and deep learning. It is strongly anticipated that more and more investigations will conduct the diagnosis and prediction of AD with neuroimaging genomics using deep learning models. Because of our goal in this review, only the diagnosis and prediction of AD with neuroimaging genomics using deep learning models have been depicted. Although in the present review we only present several research reports describing the relevant deep learning models in this application, it is strongly anticipated that deep learning models would be applied to other research areas in AD research such as dimensionality reduction, biological image analysis, and feature extraction in the near future [8].

## 8. Other Relevant Applications in Neuroimaging Genomics

In the previous sections, we describe a wide variety of research studies for the diagnosis and prediction of AD using various deep learning approaches. While this review does not intend to cover all studied that have been reported in an exhaustive manner, it nevertheless is representative of the general trend for current research in the diagnosis and prediction of AD using deep learning models. However, it is arguable that what applications could likely be considered as the focus of attention in the fields of AD research and neuroimaging genomics nowadays. The reader can refer to reviews by Medland et al. [94] and Shen et al. [95] for other relevant research studies in neuroimaging genomics using the conventional statistical approaches (e.g., multivariate regression models), which are not the focus of this review.

For example, in the context of neuroimaging genomics, a recent study proposed a latent representation learning approach for the diagnosis of AD using neuroimaging data (i.e., PET images, MRI images) and genetic variants (i.e., SNPs) [96]. Their latent representation learning approach was based on the concept of the augmented Lagrange multiplier [97], which is generally used to solve nonlinear optimization problems. Their findings showed that the model with all the three modalities (i.e., PET images, MRI images, and SNPs) outperformed any model with any combination of two modalities [96].

## 9. Conclusions and Perspectives

In brief, the present study has addressed some key issues in the aforementioned research studies, including a lack of large-scale universal/standardized benchmark databases, the absence of standardized benchmark models, without the utilization of cross-validation procedures, computer hardware limitations, without interpretability for deep learning models, a lack of open-source software platforms/packages, and the instability and the mode collapse issues for the GAN model, to name a few.

One of the contributions of the present study is that we outline the major points (e.g., models, data, tasks, databases, cross-validation procedures used, sample size, and performance) of the aforementioned research studies for the diagnosis and prediction of AD using various deep learning-based models. Second, we draw the connection between the aforementioned research studies by summarizing the similarities and differences of these investigations. Moreover, we depict some essential issues, open challenges, and emerging problems in these aforementioned research studies.

The practical implications of this research are twofold: first, it provides insights into aspects of deep learning models with neuroimaging and genomics data in AD prediction; and second, it demonstrates how deep learning models can facilitate the diagnosis and prediction of AD using neuroimaging and genomics data.

In conclusion, deep learning models with neuroimaging and genomics clarify to extend innovative techniques for the diagnosis and prediction of AD as indicated by the aforementioned findings. In the context of neuroimaging genomics, it is of great clinical importance that future prospective research investigations should target deep learning models to detect AD, which may contribute to feasible personalized medicine solutions in the fields of global health, public health, and population health. In consideration of the vital needs of novel techniques, deep learning models will be clearly developed towards the arena of AD research and neuroimaging genomics in the fields of global health, public health, and population health [98]. Therefore, we would anticipate that the current advances in disease diagnosis technologies and data-intensive healthcare science may undoubtedly take advantage of innovative deep learning models with neuroimaging and genomics for the fields of global health, public health, and population health during the next few years [99,100]. As a consequence, the general public and governments would have to address open challenges and emerging issues with significant preferences in the upcoming years [101,102,103]. In the next decade to come, the diagnosis and prediction of AD concerning deep learning models with neuroimaging and genomics would be materialized for target-specific therapeutics in personalized medicine when prospective large-scale investigations are able to extensively appraise the relevant novel neuroimaging and genomics features [104,105,106].

## Figures and Tables

**Table 1:** Relevant studies on the deep learning-based models of AD prediction.

Study	Model	Data	Tasks
Maj et al. [40]	FNNs, CNNs, RNNs	Gene expression	Predict AD risk
Lee and Lee [41]	FNNs	Gene expression	Predict AD risk
Park, J. et al. [42]	GANs	Gene expression	Predict the virtual disease/molecular progress of AD
Kim et al. [43]	Residual CNNs	Gene expression	Predict AD-specific nucleotide alteration sites (i.e., splicing sites)
Park, C. et al. [44]	FNNs	Gene expression, DNA methylation	Predict AD risk
Ju et al. [45]	Autoencoders	MRI	Predict early diagnosis of AD
Shen et al. [46]	DBNs	PET	Distinguish AD from MCI
Zhou, P. et al. [47]	Sparse-response DBNs	PET, MRI	Predict AD risk
Ning et al. [48]	FNNs	SNPs, MRI (brain measures)	Predict AD risk
Zhou, T. et al. [49]	Three-stage FNNs	SNPs, ROIs in PET, ROIs in MRI	Predict AD risk
Zhou, J. et al. [50]	CNNs	SNPs, ROIs in MRI	Predict AD risk

CNNs = Convolutional Neural Networks; DBNs = Deep belief networks; FNNs = Fully Connected Neural Networks; GANs = Generative Adversarial Networks; MCI = mild cognitive impairment; MRI = magnetic resonance imaging; PET = positron emission tomography; RNNs = Recurrent Neural Networks; ROIs = regions of interest; SNPs = Single nucleotide polymorphisms.

**Table 2:** Summary of the main points for the studies as shown in Table 1.

Study	Datasets	CV	Sample size	Performance
Maj et al. [40]	ADNI	5-fold CV	528 (247 AD/281 NC)	RNNs performed best on the adipose subcutaneous, artery aorta, and colon transverse tissues (AUC = 0.953, 0.951, and 0.946, respectively). CNNs performed best on the brain spina, thyroid, and whole blood tissues (AUC = 0.943, 0.95, and 0.947, respectively).
Lee and Lee [41]	ADNI	5-fold CV	467 (157 AD/310 NC)	FNNs did not always outperform other benchmarking models (e.g., logistic regression, L1-logistic regression, support vector machines, and random forests) due to the limited sample size (no specific AUC values).
Park, J. et al. [42]	GEO	NA	36	GANs identified cholesterol biosynthesis, which is originated during an early stage of AD and is activated by amyloid-beta production.
Kim et al. [43]	GEO	NA	NA	SpliceAI (based on the residual CNN model) identified 14 splicing sites in the *PLCG1* gene, which contains the AD-associated SNVs.
Park, C. et al. [44]	GEO	5-fold CV	696 (439 AD/257 NC)	FNNs exceeded naive Bayesian, support vector machines, and random forests (AUC = 0.797, 0.756, 0.773, and 0.775, respectively).
Ju et al. [45]	ADNI	10-fold CV	170 (91 MCI/79 NC)	The auto-encoder model outperformed linear discriminant analysis, logistic regression, and support vector machines (AUC = 0.916, 0.710, 0.765, and 0.789, respectively).
Shen et al. [46]	ADNI	5-fold CV	109 (47 AD/62 MCI)	DBNs surpassed PCA and anatomical auto-matic labeling (accuracy = 0.866, 0.795, and 0.631, respectively; no specific AUC values).
Zhou, P. et al. [47]	ADNI	5-fold CV	340 (116 AD/82 MCI/142 NC)	The sparse-response DBN-based model exceeded CNNs and DBNs for differentiating AD and normal controls (AUC = 0.87, 0.77, 0.83), MCI and normal controls (AUC = 0.79, 0.60, 0.73), and AD and MCI (AUC = 0.71, 0.60, 0.68).
Ning et al. [48]	ADNI	NA	721 (138 AD/358 MCI/225 NC)	FNNs outperformed logistic regression for differentiating AD and normal controls (AUC = 0.948, 0.945) and differentiating AD and MCI (AUC = 0.846, 0.824).
Zhou, T. et al. [49]	ADNI	20-fold CV	805 (190 AD/389 MCI/226 NC)	Three-stage FNNs surpassed support vector machines (no specific AUC values).
Zhou, J. et al. [50]	ADNI	5-fold CV	632	CNNs exceeded multivariate regression, ridge regression, group-sparse mul-ti-task regression and feature selection, and multi-task feature learning (no specific AUC values).

ACC = accuracy; AD = Alzheimer’s disease; ADNI = Alzheimer’s Disease Neuroimaging Initiative; AUC = the area under the curve; CNNs = Convolutional Neural Networks; CV = cross-validation; DBNs = Deep belief networks; FNNs = Fully Connected Neural Networks; GANs = Generative Adversarial Networks; GEO = Gene Expression Omnibus; MCI = mild cognitive impairment; MRI = magnetic resonance imaging; NA = not available; NC = normal controls; OASIS = Open Access Series of Imaging Studies; PCA = principle component analysis; RNNs = Recurrent Neural Networks; SNVs = single-nucleotide variants.

## Supplementary Materials

The following are available online at https://www.mdpi.com/article/10.3390/ijms22157911/s1, Figure S1: PRISMA flow diagram.
