# Supplementary figures and images for: Deep Learning with Neuroimaging and Genomics in Alzheimer’s Disease
Source: Int J Mol Sci. 2021 Jul 24;22(15):7911. doi: 10.3390/ijms22157911 (PMC8347529; doi:10.3390/ijms22157911)

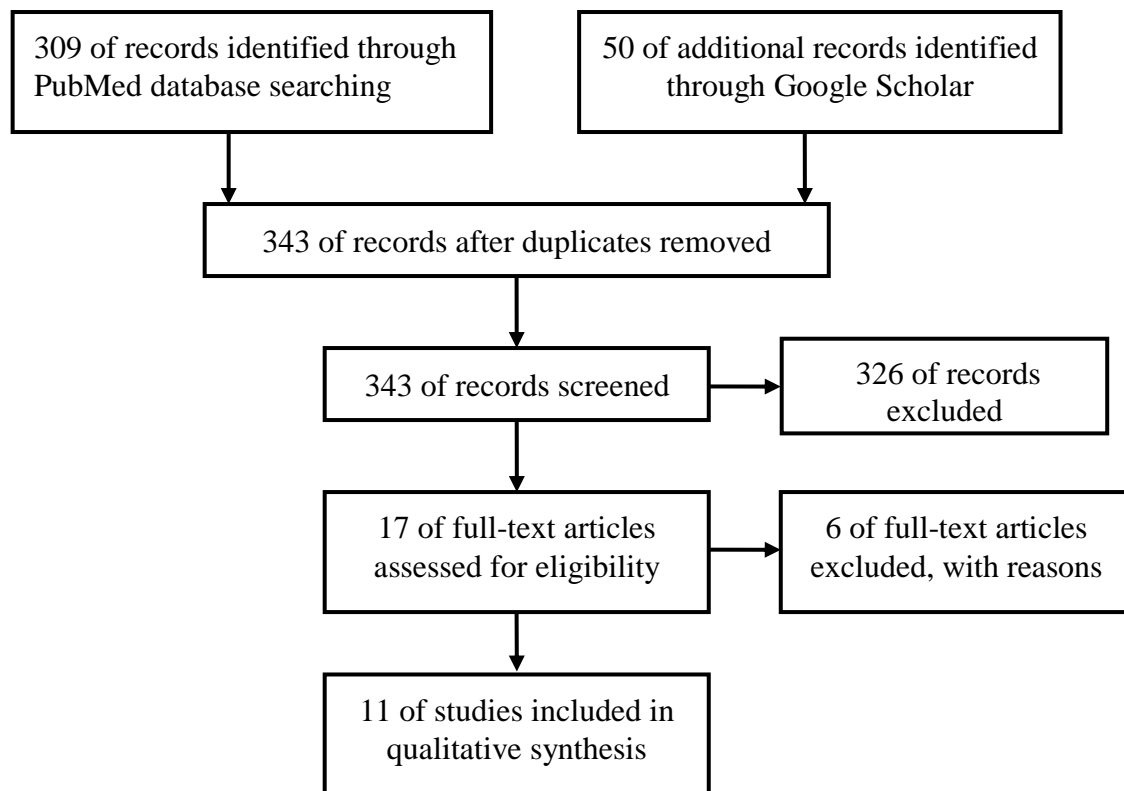

**Supplementary Figure S1.** PRISMA flow diagram.

Supplement: Supplementary file 1 [file ijms-22-07911-s001.zip › ijms-1294061-supplementary.pdf]
